# Prevalence of the Ser217Leu Variant of the ELAC2 Gene and Its Association with Prostate Cancer in Population of the Littoral Region of Cameroon

**DOI:** 10.1155/2019/5974928

**Published:** 2019-06-19

**Authors:** Alexandra Lindsey Zune Djomkam, Theodore Beyeme Sala, Clarisse Baari Memba, Dieudonné Lemuh Njimoh

**Affiliations:** ^1^Department of Biochemistry and Molecular Biology, Faculty of Science, University of Buea, Buea, Cameroon; ^2^Laquintinie Hospital, Douala, Cameroon; ^3^Allegra Foundation, Douala, Cameroon

## Abstract

*Background. HPC2/ELAC2 *has been identified as a prostate cancer (PC) susceptibility gene. Ser- Leu changes at amino acid 217 have been one of the most studied variants of this gene. Several reports have shown association of this variant with PC in samples of men drawn from families with hereditary PC and even sporadic cases.* Aim.* This study aimed at assessing this association and the prevalence of the Ser217Leu variant of ELAC2 in populations of the Littoral Region of Cameroon.* Method.* 103 PC case subjects and 80 randomly selected controls identified from the study population participated in the study. 2 milliliters of blood samples was collected from each of the consented participants and used for human genomic DNA extraction and genotyping of the ELAC2 gene by the nonenzymatic salting out and PCR-RFLP methods, respectively.* Results.* The frequencies of the wild type (SS), heterozygous mutant (SL), and homozygous mutant (LL) genotypes were, respectively, 28.2%, 49.5%, and 22.3% in prostate cancer patients and 28.8%, 67.5%, and 3.7% in controls. Comparing the LL with SS and (SL+LL) with SS showed that the presence of two copies of the L allele confers a high risk of prostate cancer as compared to the presence of only one L allele which presents no risk of prostate cancer (OR = 6.080 and 1.030, respectively). Analysis of our results also suggested an association (*P *= 0.0012) of the Ser217Leu variant with increased risk of prostate cancer.* Conclusion.* Alterations in the ELAC2 gene contribute to prostate cancer susceptibility in men living in the Littoral Region of Cameroon.

## 1. Introduction

Gene mutations have been suggested and demonstrated in some cases to be a predisposing factor for an increased risk of some common diseases including Prostate Cancer (PC). Prostate cancer ranks as the second most frequent cancer and the fifth leading cause of cancer death worldwide [1, 2]. It is the most frequently diagnosed cancer among men in most countries of the world and is the leading cause of cancer related death among men in about 46 countries, especially in the Caribbean and Sub‐Saharan Africa regions [1, 3]. Though not much is known about the etiology of prostate cancer, its high rate among men of African descent suggests ethnic and genetic predisposition though there also exist other additional risk factors for advanced prostate cancer. An examination of patterns in prostate cancer incidence and mortality across populations and over time provides insights into the role of individual risk factors and population screening behaviors in the epidemiology of the disease. In Cameroon PC is the most diagnosed cancer among men [4]. Due to inadequate infrastructure, record keeping, and resources, little is known about its actual burden on the population of Cameroon. Few studies have, however, addressed its epidemiomorphology [5] and urban specific prevalence of all male cancers [6]. Most studies suggest that genetic factors are involved in the etiology of prostate cancer [7] and in that line many loci that may harbor PC susceptibility genes have been identified during the last decades. Just recently, genome-wide association studies identified more than one hundred PC susceptibility loci though how the genetic variants at most of these loci confer disease risk remains to be determined [8]. One of such loci is the* ELAC2 *gene which was the very first to be identified as a PC susceptibility gene. The protein product ELAC2 has been suggested to play a number of different roles in the cell including the regulation of cell-cycle progression [9–11]. A number of studies on the association of the two common missense mutations (Ser217Leu and Ala541Thr) of* ELAC2* with the risk of developing PC have been carried out yielding conflicting results [12–14], most likely due to differences in environmental and lifestyle factors. Due to the marked inconsistency in findings of the association of* ELAC2* polymorphism and prostate cancer in different geographical locations and ethnicities, establishment of this association in the Cameroonian population can throw more light on this association as well as revealing new insights with regards to the management of the disease in this area. The study described herein was undertaken to determine the prevalence and association of the Ser217Leu variant of the* ELAC2* gene in prostate cancer patients of the Littoral Region of Cameroon. This could provide additional information that may potentially be exploited for early screening and diagnoses of high risk individuals for early therapeutic intervention or ease of management.

## 2. Materials and Methods

### 2.1. Ethics Concern

This study was approved by the Ethics Review and Consultancy Committee (ERCC) of the Cameroon Bioethics Initiative (CAMBIN) (Ref. No: CBI/382/ERCC/CAMBIN). Written informed consent was obtained from all participants.

### 2.2. Study Area

The study was carried out at the Laquintinie reference Hospital in Douala, Cameroon. Douala is the capital of the Littoral Region which is the economic capital of Cameroon. It is the largest, most populous, and cosmopolitan city in Cameroon. It has as geographical coordinates 04°03'N and 009°41'E [15]. The Laquintinie Hospital welcomes patients of different ethnicities from all over Cameroon.

### 2.3. Study Subjects

The study involved uniquely men who were either prostate cancer positive or healthy volunteers aged between 40 and 93 years. All PC cases enrolled in the study were confirmed and registered cases from the Laquintinie reference hospital in Douala. Healthy volunteers were enrolled following mass sensitization in the quarters. Written informed consent was obtained from volunteers who were then made to fill a structured questionnaire prior to enrolment into the study.

### 2.4. Sample Collection and Measurement of Hemoglobin Content

Two milliliters of blood from each participant was collected by venipuncture into EDTA- vacutainer tubes following strict aseptic techniques. Hemoglobin levels were determined using the URIT-12 hemoglobin and H12 hemoglobin test strips (Guangzhou Labon Medical Equipment CO., LTD). The samples were then transported well packaged on ice to the Biotechnology Unit at the University of Buea where they were stored at -20°C until required for DNA extraction.

### 2.5. DNA Extraction

Human genomic DNA was extracted from the whole blood samples using the previously described nonenzymatic salting out procedure [16]. Briefly, 900 *μ*l of TKM 1 and 50 *μ*l of 1x Triton-X were added to 300 *μ*l of heparinised blood in an eppendorf and incubated at 37°C for 5 minutes to lyse the red blood cells (RBCs). Cells were centrifuged at 8000 rpm for 3 minutes and the supernatant was discarded. The pellet was next resuspended in 900 *μ*l of TKM1 and 40 *μ*l of 1x Triton-X and centrifuged as above. This step was repeated 3 times with decreasing amount of 1x Triton-X till RBC lysis was complete and a white pellet of white blood cells (WBCs) was obtained. 300 *μ*l of TKM 2 and 40 *μ*l of 10% SDS were next added to the cell pellet and incubated for 5 minutes at 37°C. After incubation, 100 *μ*l of 6M NaCl was added and vortexed to precipitate the proteins. Cells were centrifuged at 8000 rpm for 5 minutes and the resulting supernatant was transferred into a new eppendorf tube containing 300 *μ*l of isopropanol. DNA was precipitated by inverting the eppendorf slowly and then centrifuging at 8000 rpm for 10 minutes. The supernatant was discarded and the DNA pellet obtained was resuspended in 70% ethanol to remove any excess salts. Finally the tubes were centrifuged at 8000 rpm for 5 minutes to pellet down the DNA. The DNA pellet was then air-dried, resuspended in 50 *μ*l of TE buffer, and stored at -20°C until required for analysis.

### 2.6. Amplification of the* ELAC2 *Gene

The* ELAC 2* gene was amplified from the isolated genomic DNA of all the study participants by the Touch down polymerase chain reaction (Touch down PCR) method. The missense mutation at exon 7 of* ELAC2 *(Ser217Leu) was amplified as a 231bp fragment using the forward and reverse primers 5'GGCTGTCAGCTCACCTTGTG3' and 5'GCAGAGAATTAAGAAAACGCAAGC3', respectively. These primers and PCR conditions have previously been described [17]. All primers were obtained from Inqaba Biotech industries (South Africa). Amplicons were confirmed on 2% ethidium bromide stained agarose gels.

### 2.7. Restriction Fragment Length Polymorphism Analysis of* ELAC2 *Amplicons

Digestion of the PCR products for the genetic variation of the* ELAC2 *(Ser217Leu) gene made use of the Taq alpha I enzyme and as instructed by the manufacturers with some slight modifications. 0.5 *μ*l of the enzyme, 2.5 *μ*l of the corresponding buffer, 7.0 *μ*l of nuclease free water, and 10 *μ*l of PCR product in a total volume of 20 *μ*l were incubated at 65°C for 10 minutes. Subsequently, genotypes were visualized on 2.5% agarose gel.

### 2.8. Statistical Analyses

Comparison of continuous variables between patients and randomly selected individuals was evaluated using the unpaired student t test whereas the association of categorical variables was carried out using Chi square test at a 95% confidence interval and considered significantly different at* P *< 0.05. Differences in genotype frequencies between cases and controls were tested using the standard chi-squared tests. Odds ratios were calculated using standard methods. All the above mentioned statistical analysis was carried out using GraphPad Prism Version 5.0.

## 3. Results

### 3.1. Sociodemographic Characteristics of Study Subjects

A cohort of 183 participants took part in the study and consisted of 103 prostate cancer patients and 80 randomly selected control individuals aged 40-93 years. Demographic data and information about lifestyle attitudes towards some risk factors of prostate cancer were obtained from each participant.  Table 1 summarizes the basic sociodemographic characteristics of the study population.

Prostate cancer patients were also investigated for family history of prostate cancer and 19 out of 87 respondents (21.84%) affirmed having a family history.

### 3.2. Genotyping of Ser217Leu* ELAC2* Gene

**Figure 1:**
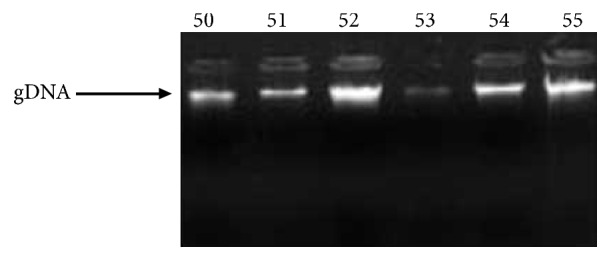
Agarose gel Electrophoregram of genomic DNA extracted from participants' blood by the salting out method. Numbers represent participants' codes.

In order to genotype for the Ser217Leu* ELAC2 *gene polymorphism, genomic DNA was isolated from participants' blood by the nonenzymatic salting out method. DNA isolates were confirmed on a 1% agarose gel stained with ethidium bromide and visualized by UV fluorescence (Gel Doc apparatus (BioRad)). The genomic DNA in all samples was successfully extracted as revealed by the presence of a band at the top of the electrophoregram (Figure 1).

**Figure 2:**
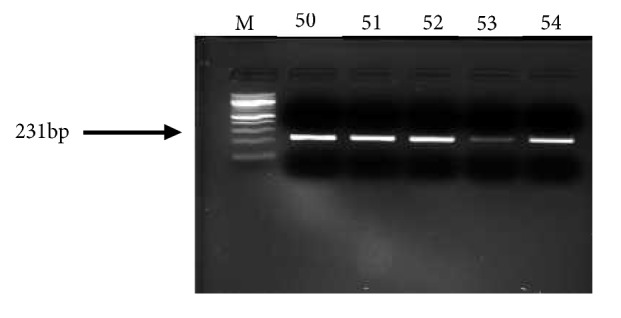
Agarose gel electrophoregram of ELAC2 gene amplified by touchdown PCR from participants' genomic DNA extracts. Lane M = 100bp molecular weight marker and lane 50-54 = DNA amplicons of participants. Numbers represent participants' codes.

The* ELAC2 *gene was next isolated from the DNA extracts of participants by the touchdown PCR method. Five microliters of each PCR product (amplicons) was ran on a 2% ethidium bromide stained agarose gel alongside a 100bp DNA ladder (BioRad). The* ELAC2 *gene was successfully isolated from all the participants' DNA extracts as a sharp band at 231bp (corresponding to the size of the* ELAC2 *gene) was obtained for each amplicon after electrophoretic analyses (Figure 2).

**Figure 3:**
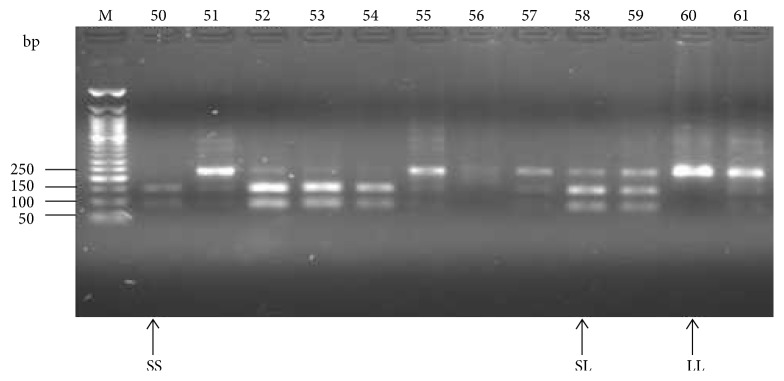
Agarose gel electrophoregram of representative results of the digestion of the ELAC2 gene with Taq*α*1. SS represents the wild-type genotype, SL the heterozygous genotype and LL the mutant genotype. Lanes 50-61 represent the ELAC2 gene digests from various participants. Lane M is the 50bp molecular weight marker. Numbers (50-61) represent various participants' laboratory codes.

To detect the Ser217Leu mutation, the PCR products of the 183 samples were digested using Taq*α*1 restriction enzyme according to the manufacturer's instruction. After analyzing the digest on a 2.5% agarose gel alongside a 50bp molecular weight marker the 3 different genotypes (wild-type (SS), heterozygous (SL), and homozygous mutants (LL)) were delineated (Figure 3).

### 3.3. ELAC2 Genotypes Frequencies and Their Association with Prostate Cancer

**Figure 4:**
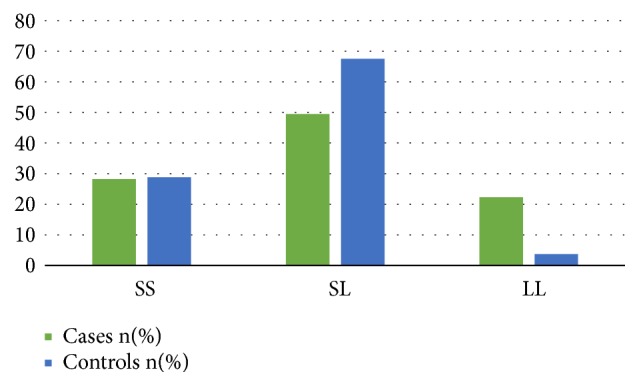
Prevalence of the different genotypes (SS, SL, and LL) of the Ser217Leu variant of the ELAC2 gene in isolates from the Littoral Region. SS= wild type, SL = heterozygous mutant, and LL = homozygous mutant. Frequencies of the genotypes SS, SL, and LL were, respectively, 28.2%, 49.5%, and 22.3% in prostate cancer cases and 28.8%, 67.5%, and 3.7% in the controls (p-value = 0.0012).

After genotyping all the 183 samples, the genotype frequencies were established (Figure 4).

The frequencies of the genotypes SS, SL, and LL were, respectively, 28.2%, 49.5%, and 22.3% in prostate cancer cases and 28.8%, 67.5%, and 3.7% in controls as seen in Figure 4. Moreover, there was a statistically significant difference in genotypes between cases and controls. However, there was no significant difference in allele frequencies between the two groups (Table 2).

The Ser217Leu mutation was high in both the prostate cancer patients and the controls. However, the frequencies of the homozygous mutants (LL) and the heterozygous mutants (SL) were relatively higher, respectively, in patients and controls.

Assessing the risk of developing PC cancer with regards to the presence of various genotypes (Table 3) showed that the presence of two copies of the mutant allele (LL) confers a high risk of developing prostate cancer (p-value = 0.0047).

We went ahead to find out if the mutant allele increased the risk of early disease onset by assessing the relationship between the alleles and the age at diagnosis (≤ 65 years and > 65 years) but found no increased risk (*p* = 0.1320; OR = 0.5748 at 95% CI). To know if this mutation might be involved in increased severity of the disease, we compared PSA levels (< 10, 10-19.99, 20-49.99, 50-99.99, and ≥ 100 ng/mL) to allele frequencies and obtained* p *value of 0.9807.

## 4. Discussion

Prostate cancer continues to have an unclear etiology though substantial evidence exists indicating that the etiology involves a combination of genetic susceptibility factors and external risk factors (environmental factors). These differences account for the variations in PC incidents around the globe. Whereas several of the risk factors are merely the result of individual choices (such as diet, exposure to UV radiation, tobacco use) some major risk factors for prostate cancer are determined and unchangeable, including age, ethnicity, and family history. Among genetic factors, several high-penetrance genetic variants have been identified based on genetic linkage and genome wide association studies as well as many other yet uncharacterized susceptibility loci. Genetic variations in genes including the* ELAC2* gene have been shown to constitute key genetic factors that predispose many populations to an increased risk of developing PC. Studies have provided proof-of-principle that some of the genetic variants of some of these genes including the* ELAC2* can serve as biomarkers of PC detection and progression as well as for the identification of high-risk individuals [12, 13]. This will facilitate the early detection of the disease and the ease of management given that most PC patients present at the hospital with clinically advanced and aggressive stages of the disease with treatment not readily affordable and available. In this study, we set out to investigate the prevalence of the Ser217Leu variant of the* ELAC2 *susceptibility gene and its association with PC in population of the Littoral Region of Cameroon. There is increased incidence of prostate cancer worldwide and especially in Africa in recent years [18, 19].

The* ELAC2* gene was genotyped in 183 participants of the study and the frequency of each genotype was determined. The frequencies of the genotypes SS, SL, and LL were, respectively, 28.2%, 49.5%, and 22.3% in prostate cancer patients and 28.8%, 67.5%, and 3.7% in controls. There was a high frequency of the Ser217Leu mutation in both the prostate cancer patients and the controls. We, however, found a significant difference between the patient and the control groups for Ser217Leu genotype frequencies in the* ELAC2* gene (*p *=0.0012). This significant difference is attributed to the great disparity between the frequency of the homozygous mutant between the cases and the controls. There was no significant difference between the individual alleles in cases and the controls. However, comparison of the LL with SS and (SL+LL) with SS showed that the two copies of the L allele confer an increased risk of prostate cancer compared to having just a single copy of the mutant allele (OR = 6.080 and 1.030, respectively). The frequencies of the S and L alleles in cases in this study were 52.91% and 47.09% which is similar to results from several studies notably those obtained by Izmirli and colleagues (52.3% and 47.7%) in 2011 [20]. However when comparing the allele frequencies of the cases to the controls, there was no significant difference (0.0710) whereas Izmirli and colleagues found a significant difference (p < 0.001). Robbins et al. also obtained a significant difference [21]. On the other hand, studies carried out by Vesprini and colleagues in 2001 [22] as well as Rökman and colleagues in 2001 [23] showed no significant difference and therefore no association between the Ser217Leu variant and the prostate cancer [22, 23]. The sample size might have been a contributing factor to these discrepancies but a number of meta-analyses studies also yielded conflicting results [12–14]. The differences in all these results could be attributed to different ethnic backgrounds and geographical locations of various study populations. There are several studies which reveal that there is correlation between prostate cancer patients and their ethnic backgrounds. This has been demonstrated, for instance, in the Caucasian, African-American, Asian, Canadian, American-Caucasians, and British populations for the Ser217Leu polymorphism in the ELAC2 gene [24, 25].

We proceeded to find out if the mutant allele increases the risk of early disease onset by testing the relationship between alleles and age at diagnosis of the cases. We found out that there was no increased risk for the mutant alleles (*p *= 0.1320; OR = 0.5748 at 95% CI). To ascertain if this mutation might be involved in increased severity of the disease, we compared PSA levels to allele frequencies. The result obtained showed that the mutation is not involved in increased severity of the disease (*p *= 0.9807).

## 5. Conclusion

Genotyping of ELAC2 gene in population of the Littoral Region of Cameroon revealed a high prevalence of the Ser217Leu variant between cases and controls. While the heterozygous mutant was the most prevalent genotype in both groups, the homozygous mutant genotype was largely prevalent in the cases than in the controls and the difference was significant. Our findings show that the presence of two copies of these mutant alleles increases the risk of PC.

## Figures and Tables

**Table 1:** Sociodemographic characteristics of study subjects.

Parameters	Cases (n = 103)	Controls (n = 80)	*P *value
Age (at enrollment)	67.80 ± 7.44	56.39 ±12.60	**<**0.0001
BMI	25.59 ± 4.21	26.82 ± 3.51	0.0676
Hb	11.42 ± 2.18	12.58 ± 2.22	0.0011
Smoking	33 (34.02%)	23 (37.70)	0.7329
Excess Alcohol intake	61 (63.54%)	51 (86.44%)	0.0028
Lack of physical exercise	41 (43.16%)	31 (52.54%)	0.3191

BMI = body mass index, Hb = hemoglobin count.

**Table 2:** Allelic frequencies of the ELAC2 gene in the study participants.

	Ser217Leu	Patients	Control	*P-*value	OR (95% CI)
		n (%)	n (%)		
Allele	S	109 (52.91)	100 (62.5)	0.0710	0.6742
	L	97 (47.09)	60 (37.5)		

**Table 3:** Risk assessment of the different genotypes.

Genotypes compared	Genotype	Cases	controls	p-value	OR (95%CI)
SL vs. SS	SL	51	54	0.4033	0.7490
	SS	29	23		
LL vs. SS	LL	23	3	*0.0047*	*6.080*
	SS	29	23		
(SL + LL) vs. SS	SL + LL	74	57	1.0000	1.030
	SS	29	23		

## Data Availability

Details of patients'/controls' sociodemographic data supporting the findings of this study are available from the corresponding author and can only be made available to researchers who meet the criteria of access due to ethical concerns (CBI/382/ERCC/CAMBIN) in order to protect patients' privacy.
